# The Role of Innate Lymphoid Cells in Immune-Mediated Liver Diseases

**DOI:** 10.3389/fimmu.2017.00695

**Published:** 2017-06-13

**Authors:** Meifang Liu, Cai Zhang

**Affiliations:** ^1^School of Pharmaceutical Sciences, Institute of Immunopharmacology and Immunotherapy, Shandong University, Jinan, China

**Keywords:** innate lymphoid cells, liver, conventional NK, liver-resident NK, liver disease, immune-mediated disease

## Abstract

Innate lymphoid cells (ILCs) are a recently identified group of innate immune cells lacking antigen-specific receptors that can mediate immune responses and regulate tissue homeostasis and inflammation. ILCs comprise group 1 ILCs, group 2 ILCs, and group 3 ILCs. These ILCs usually localize at mucosal surfaces and combat pathogens by the rapid release of certain cytokines. However, the uncontrolled activation of ILCs can also lead to damaging inflammation, especially in the gut, lung, and skin. Although the physiological and pathogenic roles of ILCs in liver diseases have been attracting increasing attention recently, there has been no systematic review regarding the roles of ILCs in immune-mediated liver diseases. Here, we review the relationships between the ILC subsets and their functions in immune-mediated liver diseases, and discuss their therapeutic potential based on current knowledge about the functional roles of these cells in liver diseases.

## Introduction

Innate lymphoid cells (ILCs) are a recently identified group of heterogeneous innate immune cells. These newly identified ILCs are distinguished from B and T cells by their thymus-independent development and lack antigen receptors encoded by rearranged genes (1). ILCs are divided into three subsets: group 1 ILCs (comprising ILC1s and conventional NK cells); group 2 ILCs (comprising ILC2s); and group 3 ILCs [comprising ILC3s and lymphoid tissue inducer (LTi) cells], based on their ability to produce type 1, type 2, and Th17 cell-associated cytokines (2). There is a striking similarity between different T helper cells and ILC subsets in terms of phenotypes and functions (3). The conventional NK (cNK) cells are also called cytotoxic ILCs because of their powerful ability to kill target cells directly (4). The other subgroups of ILCs (ILC1s, ILC2s, and ILC3s) are termed as helper-like ILCs, based on their ability to improve body defense by the secretion of cytokines (5).

Innate lymphoid cells are found at primary entry sites of pathogens, such as in mucosal surfaces of the lung and gastrointestinal system, the skin, and the liver (5). They play a vital role in tissue homeostasis, remodeling, repair of damaged tissue, and lymphoid tissue formation (6). The liver, as an important immune-tolerant organ, is known for its predominantly innate immunity and maintaining tolerance to harmless antigens (7–9). However, uncontrolled activation and proliferation of ILCs can lead to damaging inflammation (10–13). Recent studies have highlighted the potential involvement of ILC subsets in regulating liver diseases (14–16). For example, ILC1s exhibit pro-inflammatory roles in the pathogenesis of chronic hepatitis B (14). A group of IL-33-dependent hepatic ILC2s was required and sufficient for hepatic fibrosis *via* an IL-13-dependent mechanism (17). ILC3s play a protective role in murine acute hepatitis (18). Although accumulating data support potential roles for helper-like ILCs in regulating liver diseases, the molecular mechanisms deserve further investigation. In this review, we summarize the phenotypic characteristics of ILCs, their particular roles and mechanisms in immune-mediated liver diseases, and potential therapeutic interventions for liver diseases.

## Phenotypes, Functions, and Developments of Different ILC Subsets

Group 1 ILCs were defined based on their ability to produce interferon-γ (IFN-γ) and a dependency on the T-box transcription factor T-bet for their function and development, similar to Th1 cells (19, 20). Despite the similarities between ILC1 and cNK cells, they differ in several important respects. For example, ILC1s exhibit limited cytotoxicity compared with cNK cells, by expressing high levels of TNF-related apoptosis-inducing ligand (TRAIL) and IFN-γ, but low levels of granzyme B (GmB), and perforin in response to IL-12 (21). ILC1s depend developmentally on transcription factor T-bet, but not on eomesodermin (Eomes), and they do not express, or express low levels of Eomes (22, 23). By contrast, cNK cells express both T-bet and Eomes, and develop in a strictly Eomes-dependent manner, but only partially require T-bet, at least for terminal NK cell maturation (1, 22, 24). However, emerging data indicate that ILC1s have overlapping, but different, phenotypes and functions in different tissues (19). Both intestinal and hepatic ILC1s are CD49a^+^CD49b^−^Eomes^−^ and produce high amounts of IFN-γ in mice. Hepatic ILC1s exhibit stronger cytotoxic potential by expressing higher levels of GmB, perforin, CD107a, TRAIL, and FasL compared with intestinal ILC1s (23). ILC1-like cells found in the salivary gland are similar to the hepatic ILC1s in expressing CD49a and TRAIL, but are different from hepatic ILC1s in that the majority of these cells also express Eomes and CD49b, and they produce very low levels of IFN-γ (25). ILC1-like cells found in mouse breast and prostate tumors exert a similar phenotype to salivary gland ILC1s and express CD49a and CD103 (26). The tissue environments may modulate the phenotypes and function of ILC1s; however, the potential mechanism(s) that results in these differences remains unclear (1). ILC1s and cNK cells have an important role in infectious diseases. They are required for the control of *T. gondii* infection, as confirmed in T-bet-deficient mice (19). T-bet-dependent ILC1s are essential in host defense against *Clostridium difficile* or *Salmonella enterica* infections (27, 28). Hepatic ILC1s and mucosal ILC1s are involved in tumor surveillance (1).

ILC2s are defined by their ability to produce type 2 cytokines: IL-4, IL-5, IL-9, and IL-13, with GATA-binding protein 3 (GATA3) as its signature transcription factor. ILC2s develop in bone marrow and arise from a common lymphoid progenitor (CLP). ILC2s present mainly in non-lymphoid tissues, including the brain, heart, lung, kidney, skin, intestine, and uterus, while a few ILC2s were also reported in lymphoid tissues, such as spleen and liver (29, 30). ILC2s do not express lineage markers, but express MHC II molecules, c-Kit, Sca-1, IL-33R, and IL-7Rα (31–33). They maintain tissue homeostasis, relying on the expression of IL-7Rα in response to IL-7 (31). The expression of transcription factors GATA3 and RORα allows ILC2s to produce type 2 cytokines (34, 35). ILC2s express both IL-25 and IL-33 receptors, and are responsive to IL-25 and IL-33. Similar to CD4^+^T cells, as a major regulator in type II cytokine-dependent diseases (e.g., food allergies, atopic dermatitis, sinusitis, and asthma) (36), ILC2s are able to drive type 2 inflammation (37, 38) and provide protective immunity against helminths (39).

Group 3 ILCs contain NCR^+^ILC3s (NKp46^+^ILC3s in mice or NKp44^+^ILC3s in humans), NCR^−^ILC3s, and classical LTi cells (40). One of the most prominent features of group 3 ILCs is their production of Th17-associated cytokines IL-17, IL-22, and IFNγ, and RORγt as their signature transcription factor (10, 41). All of these ILC subsets develop in bone marrow, differentiate from CLP, and require IL-7 for their development (42). LTi cells contribute to the formation of lymph nodes and Peyer’s patches (43). NKp46^+^ILC3s produce IL-22 in response to IL-23, but do not produce IL-17 in mice, while NCR^−^ILC3s and LTi ILC3s can produce both IL-17 and IL-22 (44). Takatori et al. identified CD4^+^CD3^−^LTi-like cells expressing the IL-23 receptor, the aryl hydrocarbon receptor, and CCR6 (44). These LTi-like cells play important roles in the formation of secondary lymphoid tissues and host defense by secreting Th17-associated cytokines (44). A notable difference between LTi cells and ILC3s is that the differentiation of ILC3s, as well as ILC1s and ILC2s, depends on a transcription factor termed promyelocytic leukemia zinc finger (PLZF); however, LTi and cNK cells are independent of PLZF (45). LTis and ILC3s were reported to be major mediators in the pathogenesis of inflammatory bowel diseases (IBDs) (2, 46).

ILC1s, along with cNK cells, ILC2s, and ILC3s, arise from an early innate lymphoid precursor, which is generated from CLP (47). However, it is reported that the development of ILC1s is distinct from other ILC subsets, according to a study of fate-mapping mice (5). It is now becoming clear that developmental plasticity exists between ILC1s and other ILC subsets. A proportion of ILC3s acquire the ability to produce IFNγ by upregulating T-bet in response to IL-23 and IL-12, but completely lose RORγt expression. This process gives rise to ILC3-derived ILC1s (also called ex-RORγt^+^ILC3s) (48). ILC2s can also transdifferentiate toward ILC1s in the presence of IL-12 (49). Interestingly, this shift is reversible: both ILC2s- and ILC3s-derived ILC1s can revert to ILC2s and ILC3s under the influence of IL-4 and IL-23, respectively (48, 50).

**Figure 1:**
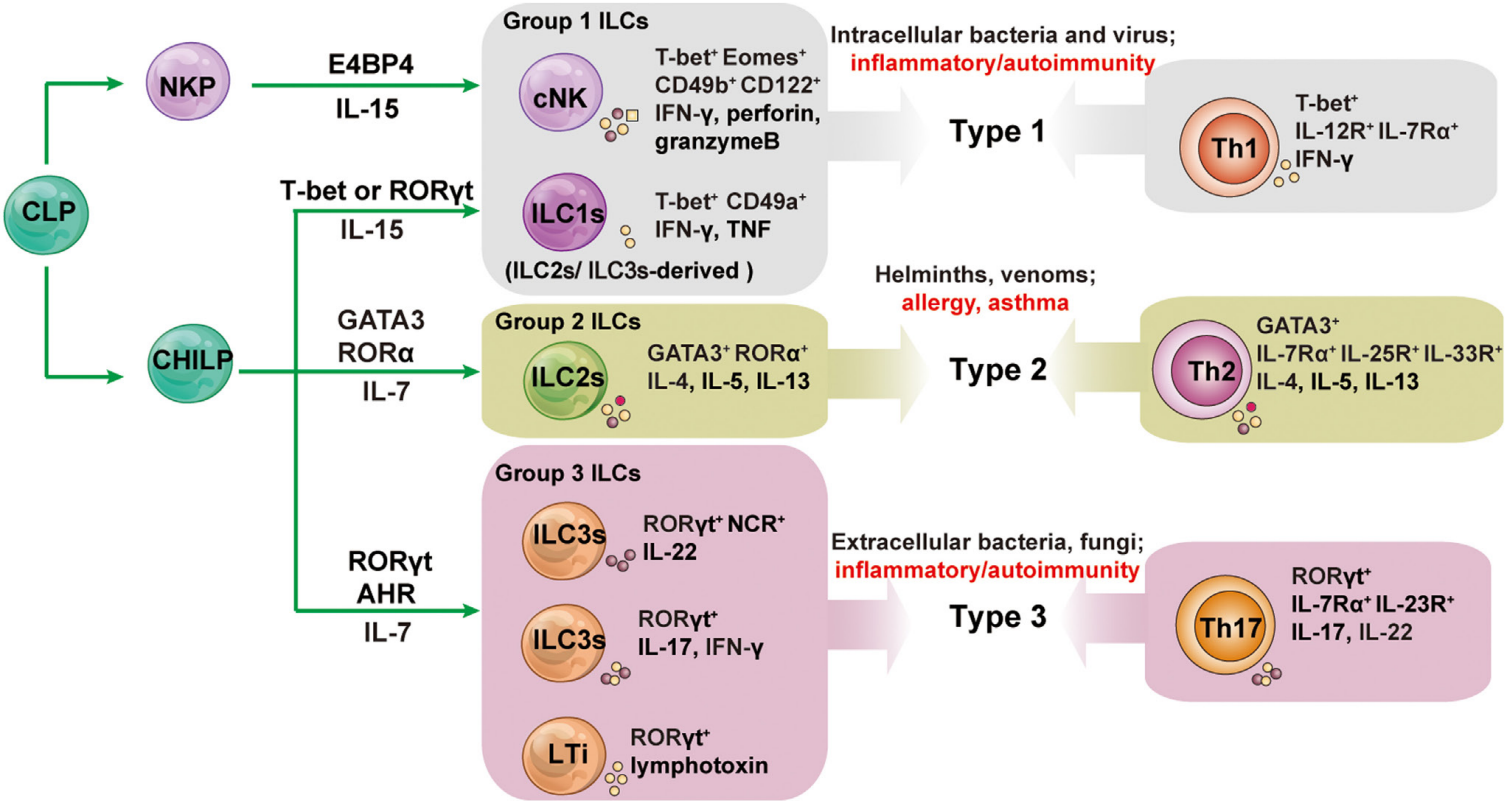
The development and functions of innate lymphoid cell (ILC) subsets and their associated adaptive lymphocyte subsets. All ILCs arise from an early innate lymphoid precursor (EILP), which is generated from the common lymphoid progenitor (CLP). ILC1s, ILC2s, and ILC3s differentiate from a common helper-like innate lymphoid precursor (CHILP) arising from EILP. Conventional NK cells (cNK) differentiate from NK cell progenitors (NKPs) arising from EILPs. Lymphoid tissue inducer (LTi) cells are a subset of innate lymphocytes that interact with stromal cells to facilitate the development of lymphoid organs. The cytokines secreted by ILCs and helper T cells promote type 1, 2, and 3 immune responses. Group 1 ILCs contain cNK and ILC1s; group 2 ILCs is the all-inclusive term for ILC2s; group 3 ILCs comprise NCR^+^ILC3s, NCR^−^ILC3s, and LTis. Unlike other helper-like ILCs, ILC1s are dependent on IL-15 signaling and not on IL-7. AHR, aryl hydrocarbon receptor; E4BP4, E4 promoter-binding protein 4 (also known as NFIL3); GATA3, GATA-binding protein 3; ID2, inhibitor of DNA binding 2; NCR, natural cytotoxicity receptor; NKP, NK cell precursor; ROR, retinoic acid receptor-related orphan receptor; Th, T helper.

The characteristics of the development, phenotype, and function of ILC subsets are summarized in Figure 1.

## The Liver as an Innate Immune Organ

The liver is the largest solid organ in the body, with two blood supplies: the portal venous system and the hepatic arterial system. It is a unique anatomical and immunological site where blood containing various antigens or microbial products circulate through a network of sinusoids and are scanned by antigen-presenting cells (APCs) and lymphocytes (51–54). The small diameter of the sinusoids and the lower systemic venous pressure result in a relatively slow flow, which lengthens the contact between APCs and lymphocytes and facilitates the clearance of harmful substances by liver-resident cells (55). The strong innate immune system in the liver distinguishes harmless from harmful molecules (56–60). The liver immune system is characterized by particularly rich in innate immune cells (e.g., Kupffer, NK, and NKT cells) (8, 60, 61). Interestingly, ILCs are detected abundantly in the liver, with the dominant ILC subsets being cNK cells and ILC1s (nearly 95% of all ILCs) (62). Although ILC2s and ILC3s in liver are quite rare (about 5% of all ILCs), the roles of these different ILCs subpopulation in liver diseases have also attracted increasing attention (17, 18).

## The Role of ILCs in Immune-Mediated Liver Diseases

### ILC1s and Immune-Mediated Liver Diseases

The liver contains abundant CD3^−^NK1.1^+^NK cells, making up about 30% of all lymphocytes in mice, while approximately 50% of hepatic NK cells are ILC1s (63, 64). Hepatic ILC1s reside primarily in the perivascular spaces surrounding the portal areas, thus they are liver-resident, whereas cNK cells migrate through the blood (63). The newly identified liver-resident NK cells were regarded as hepatic ILC1s, based on their phenotypes (expressing high levels of CD49a and TRAIL, but lacking CD49b). The development of hepatic ILC1s has similarities with that of mucosal ILC1s (19, 65). Although mucosal ILC1s and all other analyzed ILCs exhibit tissue residency akin to hepatic ILC1s, they have different phenotypes (66). Hepatic ILC1s express relatively high levels of TRAIL and FasL and exhibit stronger cytotoxic activity compared with mucosal ILC1s in the steady state (23). It is worth noting that the human liver also contains a unique liver-resident NK cell subset that constitutes nearly 50% of the entire human liver NK cells (64). It was discovered that the human liver-resident subset is phenotypically different from murine hepatic ILC1s, in that they express a high level of Eomes, but a low level of T-bet, whereas murine ILC1s are T-bet^hi^ Eomes^low^ (67). Human Eomes^hi^ NK cells are unable to leave the liver and are long-lived liver-resident cells; however, they can be replenished from the circulation during adult life, and cytokines such as IL-15 and TGF-β in the liver promote the upregulation of Eomes (68).

Hepatic cNK cells play either pro-inflammatory or anti-inflammatory role in liver inflammation (69–71). For example, the activation of cNK cells exacerbates liver injury in *Pseudomonas aeruginosa* exotoxin A or carrageenan-induced hepatotoxicity (72, 73). Hepatic cNK cells contribute to liver injury by promoting the antiviral activity of CD8^+^T in HBV infection or Con A-induced liver injury (74, 75). However, cNK cells are also reported to attenuate metabolism-induced hepatic fibrosis by regulating macrophage activation in mice (76). The role of ILC1s in liver diseases has recently been investigated. Hepatic ILC1s play potential pro-inflammatory roles and contribute to the pathogenesis of chronic hepatitis B (14). They possess memory potential and confer hapten-specific contact hypersensitivity responses upon hapten challenge (63). Notably, hepatic ILC1s contribute to maintaining liver tolerance in hepatic adenovirus (Ad) and HCV infection. NKG2A signaling in ILC1s inhibits the CXCL9 expression that is required to accumulate cNK cells, thus resulting in a loss of IFN-γ production, which is crucial to enhance antiviral CD8^+^T cell responses (77). Similarly, high expression of NKG2A was found on ILC1s in chronic HCV-infected patients. Blocking of NKG2A on ILC1s resulted in resistance to HCV *via* increasing IFN-γ production (78). Hepatic ILC1s contribute to liver regeneration after partial hepatectomy (PH) in mice. After PH, cNK cells and hepatic ILC1s produce IL-22 in response to elevated adenosine triphosphate (ATP) and IL-23 in an ATP receptor P2X1-dependent manner. IL-22 further activates STAT3 *via* binding to the IL-22 receptor, which is expressed at high levels by hepatocytes, and subsequently induces the expression of antiapoptotic (e.g., Bcl-xL, Mcl-1) and proliferation-associated (e.g., c-myc, cyclin D1) proteins (79, 80). Thus, the IL-22 produced by cNK cells and hepatic ILC1s is required for liver regeneration. cNK cells limit liver fibrosis by killing activated hepatic stellate cells (HSCs) in a TRAIL-dependent manner (81). The release of TNF-α increases the TRAIL expression on cNK cells and the TRAIL receptor on HSCs, resulting in enhanced NK cell-mediated HSC killing (82–84). With high expression of TRAIL (85), whether hepatic ILC1s interact directly with HSCs requires further investigation. These findings suggest that the cytokines (e.g., IL-22, IFN-γ) or receptors (e.g., NKG2A, TRAIL) expressed by hepatic ILC1s might serve as potential therapeutic targets in immune-mediated liver diseases.

### ILC2s and Immune-Mediated Liver Diseases

Although ILC2s are quite rare in the liver (5% or less of all ILCs), their roles in liver diseases have attracted increased attention (17, 62). A recent study demonstrated that the profibrotic effect of IL-33 is related to the activation and expansion of liver-resident ILC2s (17). The IL-33 released in response to chronic hepatocellular stress could lead to the accumulation and activation of ILC2s in the liver *via* ST2-dependent signaling. These activated ILC2s produce IL-13, a critical downstream cytokine of IL-33, which leads to HSCs activation in an IL-13Rα1- and STAT6-dependent fashion (86). These findings suggested that therapeutic modulation of IL-33-dependent ILC2s responses might alleviate chronic hepatic inflammation and fibrosis. Moreover, recently, Neumann et al. (87) reported the pro-inflammatory effect of ILC2s in a murine Con A-induced hepatitis model. They demonstrated that CD4^+^T cell-mediated tissue damage and subsequent IL-33 release enhanced the activation and expansion of hepatic ILC2s. The activated ILC2s secreted IL-13 and IL-5, which further recruit eosinophils into the liver, thus amplifying inflammatory immune responses. Depletion of ILC2s ameliorated immune-mediated hepatitis significantly (87). In an Ad-induced hepatitis model, the expression of IL-33 and its receptor ST2 increased in the liver, and IL-33 induced the expansion of ILC2s strongly. However, the expanded ILC2s exhibited a hepatoprotective role by inhibiting TNFα production (15). These findings suggested that IL-33 or IL-33-dependent ILC2s might constitute a potentially promising therapeutic candidate for the management of liver injury and viral hepatitis.

### ILC3s and Immune-Mediated Liver Diseases

ILC3s were reported to contribute to the development of IBD *via* secreting IL-17A and IFNγ in response to IL-23. Hepatic ILC3s were shown to be involved in protection or pathogenesis *via* secretion of cytokines (e.g., IL-22 and IL-17) in some liver diseases, although the percentage of hepatic ILC3s is rare (23, 62). The roles of IL-22 in liver diseases have been investigated widely (88). It is reported to prevent hepatocyte damage in carbon tetrachloride (CCl_4_), ConA, and alcohol-induced liver damage through STAT3 activation (80, 89, 90). However, during HBV infection, IL-22 exacerbated chronic liver inflammation and fibrosis by recruiting inflammatory cells into the liver (91, 92). As the main cell source of intrahepatic IL-22, ILC3s might play important roles in liver diseases. A recent study revealed a hepatoprotective role of IL-22-producing RORγt^+^NKp46^+^ cells (NKp46^+^ILC3s) in hepatic ischemia reperfusion injury (IRI) through binding to the IL-22 receptor complex, which is overexpressed by stressed hepatocytes and leads to downstream activation of STAT3 and Akt (93), which represents a therapeutic strategy in hepatic IRI by the adoptively transfer of NKp46^+^ILC3s. However, due to very rare in the liver, as in PBMCs, adoptive ILCs transfer is potentially quite difficult for humans, and effective methods have not yet been established to guarantee sufficient numbers, although it is possible to expand NKp46^+^ILC3s using cytokines (e.g., IL-15 and IL-23) before transfusion (94, 95). ILC3s are the main resource of IL-22 in CCl_4_-induced acute hepatitis and play a partially protective role in the hepatic immune response (18). Similarly, mucosal ILC3-derived IL-22 protects the intestinal epithelium from invasion of bacteria by inducing the production of the antimicrobial peptides-α and β-defensin by activating STAT3 (96). In addition, HBV induces APCs to produce IL-23, which contributes to liver damage *via* IL-17 production achieved by activating IL-23 receptor-expressing Th17 cells (97). Determining whether the IL-23 receptor-expressing ILC3s induce liver damage in HBV-induced hepatitis is worthy of further exploration. Whether hepatic ILC3s exert a protective or pathogenic role, and how the balance between these two seemingly paradoxical functions is maintained, might depend on the tissue microenvironment and inflammation status in different diseases. It is worth noting that the alteration in mucosal ILCs may impact on liver function *via* promoting the peripheral dissemination of commensal bacteria. ILCs are found essential to limit the peripheral dissemination of commensal bacteria *Alcaligenes*. Depletion of ILCs results in chronic systemic inflammation, which are associated with the progression of chronic HCV infection (98). It is suggested that alterations in ILCs and their cytokines in other tissues, particularly the gut, may have impact on the liver function and disease pathogenesis.

## Conclusion and Perspectives

**Figure 2:**
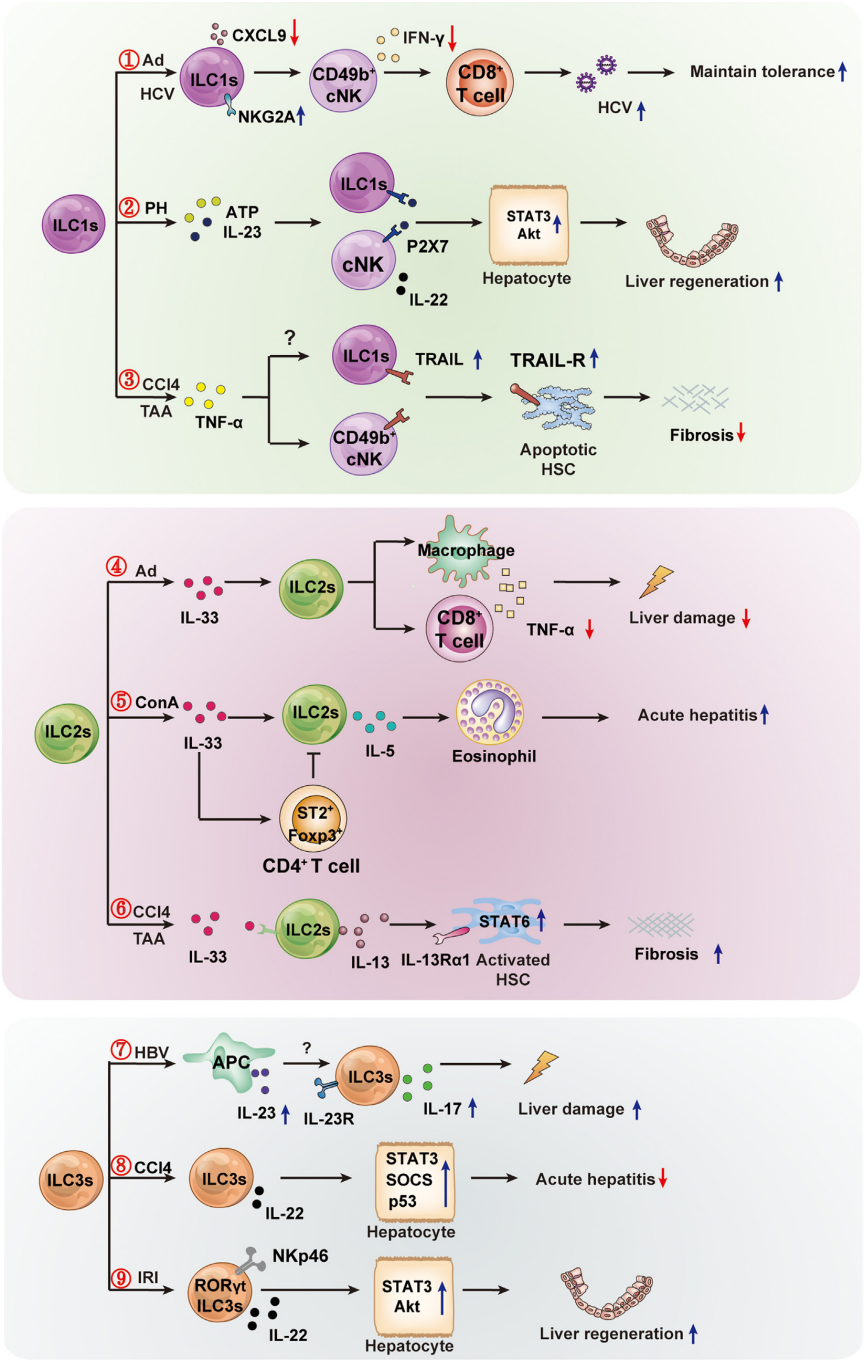
The protective or pathogenic roles of innate lymphoid cells (ILCs) in liver diseases. The hepatic ILC subsets are involved in the immune regulation of liver diseases (viral hepatitis, mechanical liver injury, and fibrosis). ① In hepatic adenovirus (Ad) or HCV infection, hepatic ILC1s play an important role in maintaining liver tolerance. Hepatic viral infection increases NKG2A expression on ILC1s, and NKG2A signaling in ILC1s inhibits CXCL9 expression, which is required for the accumulation of IFN-γ^+^CD49b^+^ NK cells (cNK cells). This, in turn, results in the loss of IFN-γ production, which is crucial for the enhanced priming of CD8^+^ T cells. ② Hepatic ILC1s and cNKs contribute to liver regeneration. cNK cells and ILC1s produce a high level of IL-22 in response to elevated adenosine triphosphate (ATP) and IL-23 in an ATP receptor P2X1 (P2-type nucleotide receptors)-dependent manner; IL-22 promotes hepatocyte growth *via* activation of the STAT3 pathway. ③ Hepatic cNK or ILC1s limit liver fibrosis. The inflammatory cytokine TNFα increases expression of TNF-related apoptosis-inducing ligand (TRAIL) on cNK cells, and then enhances cNK cell-mediated hepatic stellate cell (HSC) killing. The high expression of TRAIL may lead to hepatic ILC1s having similar effects to the killing of activated HSC, thus limiting liver fibrosis. ④ In Ad-induced liver inflammation, ILC2s exhibit a hepatoprotective role. The expression of IL-33 and its receptor ST2 in the liver are increased, and the ILC2s expand in response to IL-33 and limit liver injury by suppressing TNFα production in hepatic T cells and macrophages. ⑤ In Con A-induced hepatitis, hepatic ILC2s are activated and expanded in response to IL-33, further amplifying inflammatory immune responses *via* IL-5-mediated recruitment of eosinophils. The inflammatory activity of ILC2s might be regulated by IL-33-expanded CD4^+^Foxp3^+^ regulatory T cells (Treg). ⑥ In CCl_4_-or thioacetamide (TAA)-induced chronic hepatocellular stress, IL-33 activates ILC2s, producing IL-13, leading to HSC activation through IL-13Rα1- and STAT6-dependent signaling. ⑦ Hepatitis B virus infection induces IL-23 production by antigen-presenting cells, and the increased IL-23 contribute to liver damage *via* IL-17 production, possibly by activating the IL-23 receptor-expressing ILC3s. ⑧ In CCl_4_-induced acute hepatitis, the IL-22-producing ILC3s inhibit liver injury *via* IL-22 production. ⑨ In the pathogenesis of hepatic ischemia reperfusion injury (IRI), RORγt-expressing NKp46^+^ cells are capable of ameliorating hepatic IRI in an IL-22-dependent manner.

Recent research has made significant progress in determining the function of ILCs in liver diseases. Notably, different ILCs subpopulations might exhibit different roles in the same or different liver diseases. Even the same ILC subsets might play opposite roles in different liver diseases. These seemingly paradoxical effects might depend on the inflammation state and tissue microenvironment of different diseases. The protective or pathogenic roles of ILCs in liver diseases are summarized in Figure 2. Nevertheless, their strategic location at mucosal barriers or in liver enables ILCs to play essential roles in the maintenance of immune homeostasis by balancing destructive immunity and protective responses. It is clear that excessive activation of ILCs could lead to chronic pathologies (e.g., inflammation, liver injury, or fibrosis). Further research is needed to clarify the precise protective or pathogenic mechanisms of different ILCs in liver diseases. However, the existing findings have suggested that ILCs and their related effector molecules can be used as targets for diagnostic and therapeutic strategies. Targeting the activator or effector molecules of hepatic ILCs might be an effective treatment for liver diseases. For example, thymic stromal lymphopoietin, IL-25, and IL-33 are important activators of ILC2s, and simultaneous disruption of the three factors successfully alleviated IL-13-dependent liver inflammation and fibrosis during chronic *Schistosoma mansoni* infection (99). In view of the roles of ILC1s in maintaining tolerance to Ad or HCV infection in a NKG2A-IFNγ-DCs axis-dependent manner, it is suggested that blocking NKG2A on ILC1s in parallel with the use of a hepatic-resident DCs-targeted vaccine might constitute a novel strategy to induce sustainable CD8^+^T cell responses against hepatic pathogens (77). In addition, therapeutic adoptive transfer of hepatoprotective ILCs (e.g., IL-15 + IL-23-expanded NKp46^+^ILC3) or exogenously providing their effector molecules (e.g., protective IL-22) may also offer a promising strategy to prevent liver injury, excessive inflammation, or promote liver regeneration. Further studies are needed to improve our understanding of the distinct roles of each ILC subpopulation in immune-mediated liver diseases and to identify valid ILC-based targets for therapeutic intervention.

## Author Contributions

All authors listed have made substantial, direct, and intellectual contribution to the work and approved it for publication.

## Conflict of Interest Statement

The authors declare that the research was conducted in the absence of any commercial or financial relationships that could be construed as a potential conflict of interest.
